# Ethical challenges in qualitative sociology: a systematic literature review

**DOI:** 10.3389/fsoc.2024.1458423

**Published:** 2024-09-25

**Authors:** Carla Scheytt, Jessica Pflüger

**Affiliations:** Institute of Sociology, Faculty of Social and Political Science, University of Innsbruck, Innsbruck, Austria

**Keywords:** research ethics, reflexivity, ethical challenges, no harm, informed consent, confidentiality, anonymization, voluntary participation

## Abstract

Qualitative researchers often encounter ethical challenges during their research process. Due to the large number of papers in which researchers reflect on specific and various ethical challenges within their projects, it proves difficult to keep track of them. To capture these reflexive practices, we conducted a literature review of 72 papers in sociology. Our review shows who reflects on research ethics and when and where such reflections occur. We identify 11 ethical issues that sociologists reflect on. Some issues address the challenges of implementing established ethical principles, such as (1) informed consent, (2) voluntary participation, (3) avoiding harm, (4) anonymization, and (5) confidentiality. Others go beyond these principles and refer to (6) the relationship between researchers and participants, (7) power asymmetries, (8) protecting yourself as a researcher, (9) deviant actions, (10) covert research, and (11) leaving the field. Our findings help researchers gain an overview of ethical challenges, enhancing their reflexivity.

## 1 Introduction

Our research is situated within a broad international discourse on research ethics in qualitative research. This discourse underscores the fact that ethical challenges can manifest at every stage of the qualitative research process (Blee and Currier, 2011; Guillemin and Gillam, 2004; Iphofen, 2020a; Miller et al., 2012; Roth and Von Unger, 2018; Tolich and Tumilty, 2020). In this context, our literature review serves as a crucial tool for understanding and navigating these challenges.

Those ethical challenges are often linked to ethical principles, including harm reduction, informed consent, voluntary participation, anonymity, and confidentiality (American Sociological Association, 2018; British Sociological Association, 2017; DGS und BDS, 2017; European Commission, 2013). Researchers face the challenge of transforming abstract and sometimes conflicting ethical principles into practice. Those principles are formalized in codes, and ethical review boards require their implementation; even though the need for ethical review boards is heavily debated (Allen, 2008; Christians, 2003; Haggerty, 2004; Hammersley, 2009; Heimer and Petty, 2010). For example, researchers must decide what they understand by informed consent (e.g., ensuring participants are wholly or partly aware of the study’s purpose through written and/or oral consent, viewing consent as a circular process, etc.) and how to implement this in the project, adapted to the specific context.

However, there is no consensus that ethical issues, challenges, and moments should be understood solely as a set of specific ethical principles (Bell and Willmott, 2020; Edwards and Mauthner, 2012; Hammersley, 2015). In their daily research practice, qualitative researchers have to deal with so-called “ethically important moments” (Guillemin and Gillam, 2004). Such moments may be “difficult, often subtle, and usually unpredictable situations that arise in the practice of doing research” (Guillemin and Gillam, 2004, p. 262). They can relate to the practical implementation of ethical principles and more general issues, such as the “larger role of social science research” (von Unger, 2021, p. 192). Due to the complexity, uncertainty, and contextual embeddedness of ethical challenges, researchers, therefore, emphasize the development of an ethically reflective attitude (Guillemin and Gillam, 2004; von Unger, 2021; Warin, 2011).

Many researchers from different fields followed this call for an ethical reflexive stance and have impressively shown how they deal with “ethically important moments” in practice (see examples below). Researchers address specific ethical challenges in their projects and contexts in these contributions. Consequently, many publications highlight qualitative social research’s situation- and context-specific ethical challenges. Qualitative sociologists participate in these debates, as sociology is a field where methodological discussions are particularly lively (Aspers and Corte, 2019). We believe that this welcome movement toward greater reflexivity in research ethics would benefit from an *overview of ethical challenges* at this time. Therefore, we pose and explore the following question: What ethical challenges do qualitative researchers in sociology reflect on? We believe that answering the question and examining challenges can enhance one’s “ethical reflexivity” (von Unger, 2021) and help researchers to anticipate potential challenges.

To this end, we conducted a systematic literature review (Hannes and Macaitis, 2012; Thunberg and Arnell, 2022). There are broad and long-lasting discussions on diverse ethical issues in the methodological literature (Fine, 1993; Hammersley and Atkinson, 2007; Iphofen and Tolich, 2018; Ryen, 2010; Wiles, 2013). Addressing ethical challenges in the research process is (fortunately) nothing new. However, as we will demonstrate, the past two decades have notably increased reflexive journal articles on ethical challenges. These reflexive articles and the existing methodological literature provide a valuable perspective for understanding ethical challenges. Nevertheless, this body of reflexive research is widely dispersed and published over an extended period in various social science and sociological journals, making a systematic overview particularly helpful.

We acknowledge that employing a systematic literature review as a method may not fully align with specific characteristics of qualitative research, such as openness and in-depth data analysis. Furthermore, researchers address ethical challenges through various avenues, including publications, scientific presentations, discussions with colleagues, grey literature, unpublished memos, and field notes. Some sociologists may encounter ethical challenges in the field without adequate opportunities to discuss or publish these issues.

Despite these limitations, the method of systematic literature analysis is especially beneficial for compiling a comprehensive data set on a specific topic—in our case, ethical challenges. By analyzing numerous journal papers, we can provide an insightful overview of ethical challenges from different perspectives in various research fields and using different methodologies. Our paper is particularly valuable for a broader sociological audience conducting qualitative research unfamiliar with the literature’s complex and somewhat scattered state regarding ethical challenges. For this audience, our overview offers opportunities for further engagement with the diverse ethical issues they may encounter in the research process.

By analyzing 72 papers on research ethics and qualitative social research in sociology, we identified 11 key ethical challenges. However, these results are also relevant beyond sociology in disciplines in which qualitative methods are used, such as anthropology, education, and political sciences.

To show which ethical challenges are reported in sociological publications, we proceed as follows. First, we present our methodological approach and describe how we collected, screened, and analyzed the data in this literature review. Then, we offer the results. We show who publishes where and when on research ethics in our sample. We coded the referenced challenges into 11 issues of ethical challenges by deductively applying theoretically developed categories based on ethical principles (e.g., informed consent, confidentiality, and anonymization), and inductively developing data-driven categories (e.g., dealing with power asymmetries, conducting covert research, protecting the researcher). This combination of deductive and inductive categories, oriented by what researchers themselves define as ethical challenges, enables us to show the variety of ethical challenges researchers reflect on. We round out the argument by summarizing the most important results, discussing the limitations of our contribution, and identifying opportunities for future research.

## 2 Method: systematic literature review

We conducted a systematic literature review to determine what ethical challenges are relevant for qualitative sociological researchers (Hannes and Macaitis, 2012; Kunisch et al., 2023; Thunberg and Arnell, 2022). We aimed to explore ethical challenges that were regularly raised. To understand this, it is essential to learn more about who is publishing about research ethics, where, and in what (geographical) context. This seems helpful in keeping track of the multitude of situation- and context-specific ethical challenges.

### 2.1 Data collection

We collected papers through a database search of the Web of Science Core Collection, which contains different databases, including the Social Science Citation Index (SSCI).^1^ The Web of Science (WoS) is acknowledged as a reliable indexing tool for scientific literature (Boyack et al., 2005), including social science journals. We focused on WoS because it is the largest and most comprehensive database, so it has already been successfully used for similar literature reviews. We did not include any subject-specific databases, such as PubMed for biomedical research. We chose a database search method to incorporate various sociological and social science journals; reflective papers on research practices may appear in different journals (methodological journals, general sociology journals, special sociology journals, etc.).

Our search included sociological papers (WoS category “sociology”) that use qualitative methods (keywords: qualitative, interpretative, observation, interview, biographical, narrative, focus group, grounded theory, ethnography, phenomenological, case study, participatory, action research) and address ethical issues (keywords: research ethics, ethical dilemma, confidentiality, anonymization, informed consent, beneficence). The keywords were searched with all possible grammatical endings. To have as precise a sampling as possible, we did not include papers with a mixed method approach, even if they contain qualitative elements. For feasibility reasons, only English papers published in peer-reviewed journals were included. There were no time restrictions on publication dates. The search was carried out in July 2022 (12.07.2022).

### 2.2 Screening

**Figure 1:**
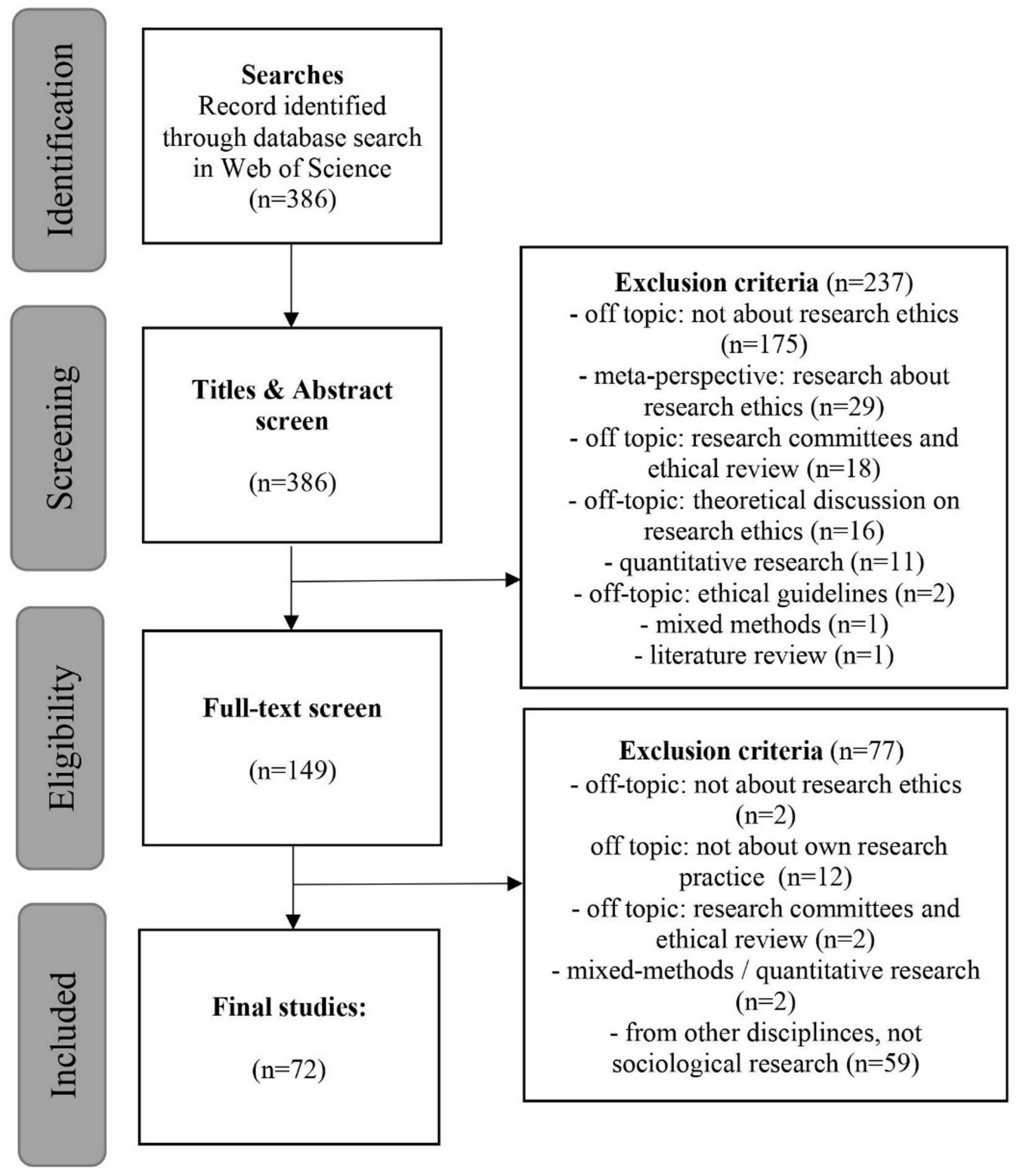
Flowchart: search strategy and screening (for the template, see Depraetere et al., 2021; Li and van den Noortgate, 2022).

Our database search yielded a number of 386 possible papers. For these papers, we conduct an initial screening (title, abstract, keywords). Our inclusion criteria were that *sociologists* write about their *research practice* and *ethical issues* in the papers. After this preliminary screening, 149 papers remained in our sample. The reasons for the exclusion can be seen in the flowchart below (Figure 1). In a second, and this time full-text screening, all papers were read and sorted out if necessary. Of the 149 papers, an additional 77 papers were eliminated in this step. The reasons for this can also be seen in the flowchart.

### 2.3 Data analysis

To conduct the analysis, we coded each of the 72 studies deductively and inductively using qualitative content analysis (Krippendorff, 2019; Kuckartz, 2018; Mayring, 2015). Deductive categories included *authors*, *institutional affiliation*, the *country* where the research was conducted, *publication year*, *methods*, *subfields*, *research objects,* and the *ethical challenges related to known principles* (e.g., gaining informed consent, see below). The code *issues of ethical challenge* was then inductively differentiated with sub-codes (e.g., *protecting yourself as a researcher*, see below). In the coding, we followed what the researchers presented as an ethical challenge. For example, we coded the *protecting yourself as a researcher* if this was mentioned in relation to research ethics. As ethical issues are often complex and diverse in the respective projects, we usually coded more than one ethical challenge per paper. Due to the large number of rather specific inductive categories that occurred only occasionally (i.e., coded 1 to a maximum of 10 times; e.g., *research used by third parties, ownership of data*, etc.), we have to limit the following presentation to the (11) main issues that occurred most frequently (coded between at least 25 and up to 100 times). We present the most mentioned challenges in this paper because those challenges arise not only in single cases but in different research projects located in various fields, carried out with diverse methods, and by multiple researchers. We therefore believe that the presented challenges are of relevance for the readership.

By selecting and limiting the topics of ethical challenges in this way, we acknowledge that we cannot provide an overview of *all* ethical challenges researchers consider. Additionally, our database has limitations (see also 4.2.). Therefore, we do not claim that our results can be generalized to all qualitative sociologists globally.

## 3 Results

### 3.1 Ethical challenges in sociology: the setting

The 72 papers we analyzed were published by 107 authors (female: *n* = 81, 75.70%; male: *n* = 26, 24.30%). Based on our data, this gendered authorship cannot be explained, but other studies point to a systematic relationship between gender and choice of method in sociology. Qualitative methods have long been known to be significantly more common among female than male scholars who publish in major sociological journals (Grant et al., 1987). Moreover, there is growing evidence that not only method choice but also topic choice is gendered in sociology (Larregue and Nielsen, 2023). Regarding *institutional settings, most researchers are affiliated* with the United Kingdom (*n* = 27) and the United States (*n* = 21). Most authors (73 of 77) work in countries of the so-called global north (see Table 1).

**Table 1:** Institutional affiliation of researchers.

Country	Amount
UK	27
USA	21
Sweden	4
South Africa	4
Australia	4
Canada	3
Norway	2
New Zealand	2
Denmark	2
Belgium	2
Other countries	6
Total	77

This dominance of the global north in publishing research ethics challenges in WoS-listed journals is also evident in the c*ountries/regions where the research was conducted*. Again, we find a focus on the UK (*n* = 21) and the United States (*n* = 11).

**Figure 2:**
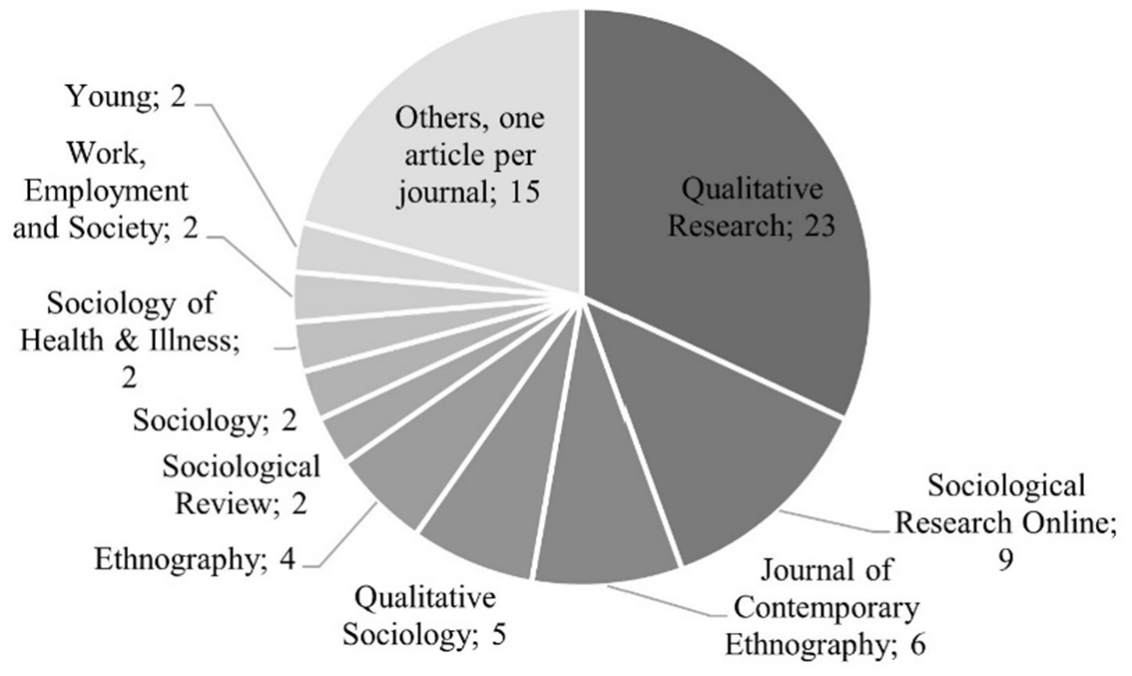
Numbers of papers in the various journals.

Our restriction to English-language journals (see Figure 2 below) may have contributed to this overrepresentation (see also 4.2). It may also be understood as reflecting a known dominance of Western sociologists in WoS publications and those who submit to WoS publications (Delamont and Atkinson, 2014).

In Figure 2, we illustrate the var*ious journals* in which the papers were published.

Overall, we observe a wide distribution of papers related to ethical challenges, analyzing publications from 25 journals. Among them, *Qualitative Research* led the list of journals with the most publications reflecting research practice. About one-third (32%) of the papers were published in *Qualitative Research.* Another 12.5% of the papers appeared in the journal *Sociological Research Online* and about 8% in the *Journal of Contemporary Ethnography*. Hence, the analyzed papers spread across journals focused on social science methods, sociological journals, and specialized fields within sociology. This seems unsurprising since methodological reflection is frequently carried out in substantive papers.

**Figure 3:**
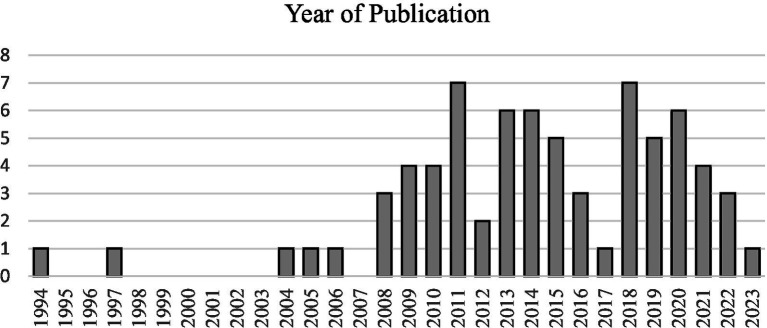
Year of publication.

Regarding the *year of publication*, most papers appeared in the 2010s (see Figure 3). Only a fraction of the 72 papers appeared before 2008 (*n* = 5).^2^ The reasons for this may be manifold. We suspect a connection to an intensification of reflection around social science research practice in general (e.g., Camic et al., 2011), and a “reflexive turn” in qualitative research (Lumsden, 2017; Venkatesh, 2013).

The *methods* used by the researchers focus on ethnographic research (44 of 72 papers) and interview research (35 out of 72 papers; see Table 2 below). This also seems unsurprising, as these are the most established methods in qualitative social research (Denzin and Lincoln, 2003; Flick et al., 2004; Seale et al., 2010). Because ethnography as a methodological approach usually involves more than participant observation, we coded those papers twice when the researchers explicitly reported that they also used interviews (same for case studies and participatory research).

**Table 2:** Use of methods.

Methods use	Amount
Ethnography and Participant Observation	54
Qualitative interviews	56
Document analysis	15
Multi methods/triangulation	19
Visual data	7
Participatory research	7
Case study	4

**Figure 4:**
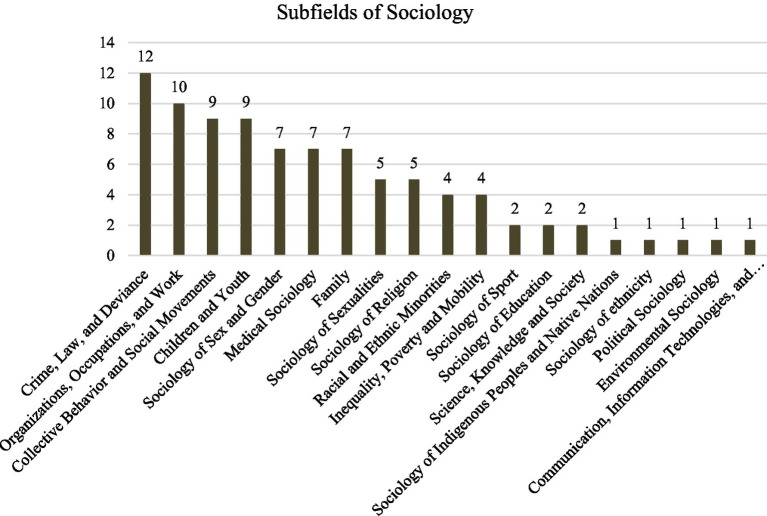
Subfields of sociology.

Finally, we want to show the *subfields of sociology* to which the papers can be assigned. We have based our coding on the American Sociological Association (ASA) sections.^3^ An overview of the specific sociologies can be found in Figure 4.

This shows that although research ethics issues are discussed in the broad field of sociology, there are nevertheless particularly pronounced reflections in two fields: the most significant number of papers comes from *criminal* and *organizational sociology*. We attribute this to the objects studied there, which entail particular challenges and reflection requirements. For example, data protection is paramount in formal organizations with strict hierarchies and potentially strong sanctions for the participants.

### 3.2 Overview of ethical challenges

Our study is particularly interested in the challenges addressed regarding research ethics in qualitative sociology. We focus on the challenges elaborated on most frequently in our sample. We present them based on the 11 key issues we discovered in the coding process (see Table 3). These issues describe the various ethical challenges that qualitative researchers in sociology reflect on in the analyzed publications. They refer to challenges linked to transforming widely known ethical principles^4^ into research practice and to challenges beyond them. (Deductive) issues related to ethical principles include (1) *gaining informed consent*, (2) *ensuring voluntary participation*, (3) *avoiding harm*, (4) *anonymizing the data*, and (5) *ensuring confidentiality*. Challenges beyond this are presented along the following issues: (6) *managing the relationship between researchers and participants*, (7) *considering power asymmetries*, (8) *protecting yourself as a researcher*, (9) *dealing with deviant actions*, (10) *conducting covert research*, and (11) *leaving the field*.

**Table 3:** Coding ethical challenges.

	Gaining informed consent	Ensuring voluntary participation	Avoiding harm	Anonymizing data	Ensuring confidentiality	Managing the relationship	Negotiating power asymmetries	Protecting yourself as a researcher	Dealing with deviant actions	Conducting covert research	Leaving the field
Angotti and Sennott (2015)	X		X	X	X						
Anspach and Mizrachi (2006)	X		X		X	X			X		
Askanius (2019)		X	X			X		X			X
Au and Marks (2013)	X	X		X							
Baarts (2009)						X					
Baird (2018)			X			X		X	X		
Barnard (2005)			X			X					
Barton (2011)	X	X	X	X	X	X	X	X			
Bernstein and Friedman (2013)			X			X	X	X		X	
Bjørnholt and Farstad (2014)	X		X	X	X						
Braye and McDonnell (2013)					X		X				
Britton (2020)			X			X	X			X	
Brosens et al. (2015)		X		X	X	X	X	X			X
Caine et al. (2009)	X										
Calvey (2008)									X	X	X
Cordner et al. (2012)	X										X
Donnelly (2014)	X					X				X	
Einwohner (2011)	X		X	X	X	X		X			
Elliott and Roberts (2020)			X	X	X	X					
Elsrud et al. (2016)	X	X		X						X	
Exley et al. (2018)					X						
Gabb (2010)	X			X	X						X
Gerrard (2021)	X			X		X		X			
Gonzalez-Lopez (2010)						X		X			
Gonzalez-Lopez (2011)	X	X	X			X	X	X			X
Grønning (1997)		X									
Guenther (2009)			X	X	X						X
Hennell et al. (2020)	X		X	X	X			X	X	X	
Jacobs (2004)			X			X		X			X
James (2013)	X	X			X	X	X		X		X
Kostet (2021)							X	X			
Kremakova (2014)	X	X	X	X		X	X	X			X
Laube (2021)	X					X				X	
Lillie and Ayling (2021)	X		X	X		X	X	X			
Lohmeyer (2020)		X			X				X		
Lumsden (2013)				X	X	X		X	X		X
Mah (2014)	X					X	X				
Mäkinen (2016)						X	X	X			
Mare (2017)	X		X	X	X						
McKenzie (2009)	X	X				X				X	
Meisel (2008)		X	X		X		X	X			X
Morrison and Sacchetto (2018)			X			X	X				
Nash and Moore (2019)	X		X		X	X		X	X		
Nielsen (2010)					X	X		X	X		
O'Connor and Goodwin (2013)	X		X	X	X						X
O'Donnell Goldensher (2022)			X	X	X				X		
Okyere (2018)	X		X								
Paoletti (2014)	X		X	X	X						X
Parvez (2018)						X	X				
Pechurina (2014)	X			X			X				
Perez (2019)	X	X	X			X	X			X	
Plankey-Videla (2012)	X	X		X	X	X	X	X			
Ramirez-i-Olle (2019)						X					
Reich (2015)	X	X			X	X		X			
Ricciardelli (2022)	X				X	X	X	X			
Rupp and Taylor (2011)			X	X		X	X		X		X
Russell and Barley (2020)	X	X	X	X		X	X				X
Saunders et al. (2015)				X	X						
Scarce (1994)			X		X			X	X		
Shaw (2011)			X			X		X			
Shaw et al. (2020)					X	X		X	X		
Stein (2010)				X	X	X	X			X	
Subramaniam (2023)				X	X	X	X	X			
Swartz (2011)			X			X	X				
Tilley and Woodthorpe (2011)			X	X							X
van Dijk (2015)	X	X	X		X	X	X	X			
Wackenhut (2018)	X		X	X	X			X			
Ward (2008)	X	X				X	X	X	X	X	X
Wills et al. (2016)	X			X							
Wilson (2018)			X	X							
Winfield (2022)		X		X	X		X				
Yusupova (2019)			X	X		X		X			X

The ethical issues we encounter are not isolated; they are interconnected and cannot be sharply delineated. For instance, issues of anonymization and confidentiality are often intertwined with avoiding harm. Anonymization of data, for example, can ensure that no harm is being done to participants. The question of covert and semi-covert research is inevitably linked to questions of informed consent and voluntary participation; informed consent and voluntary participation are interdependent, and so on.

Accordingly, and as the following table (Table 3) shows at first glance, many papers reflect not just one ethical challenge but several.

Despite these complex interrelationships, overlaps, and limitations, we consider it useful to distinguish the different issues for analytical reasons. We, therefore, proceed with the individual presentation of the 11 issues that make up our codes. To capture the breadth of ethical challenges, we must necessarily sacrifice depth. This also means that we cannot go into detail on all 72 papers analyzed.

In the following, we present the ethical issues, highlighting the most critical aspects underpinned by the current state of research. It is impossible to mention each paper and each challenge individually. Still, we will show the most important subcategories of each ethical issue with examples, allowing us to provide an illustrative overview of ethical challenges. The examples and references to the various papers provide an introduction to an in-depth examination of the respective ethical challenges. We see the following overview as an invitation for readers to engage more closely with the paper, especially where ethical challenges are discussed, and the qualitative scholars describe how they dealt with and resolved or, in some cases, endured ethical dilemmas. As we have said before, because of the characteristics of qualitative research, unforeseen ethical challenges can always arise. Nevertheless, examining existing knowledge is always helpful in developing an ethical reflexive stance.

#### 3.2.1 Gaining informed consent

Informed consent is often discussed as a fundamental principle of research ethics, linked to the underlying values of respecting autonomy and privacy. As a principle, it is highly debated among qualitative researchers since it is a central part of regulatory ethics (Christians, 2003). Researchers, especially those with an ethnographic background, criticize regulatory requirements for informed consent as unworkable and restrictive (Haggerty, 2004; Hammersley, 2009, 2015; Heimer and Petty, 2010). Despite those critiques, qualitative researchers emphasize that informed consent is an interactive and ongoing process (Guillemin and Gillam, 2004; also: American Sociological Association, 2018; British Sociological Association, 2017) and cannot be reduced to simply “signing a form” (Heimer and Petty, 2010, p. 612). Informed consent requires participants to understand the study and what they are agreeing to. Our results identify multiple challenges that correspond to this broader discussion surrounding informed consent:

In our sample, several authors explicitly point out that they understand *informed consent as an ongoing process*. For example, James (2013) explains in his prison research that inmates were repeatedly asked for consent. Wills et al. (2016) also describe that conversation regarding informed consent was repeatedly explored with participants in the research process, also Donnelly (2014) describes this regarding their participants, who were often drunk during field visits. Both Nash and Moore (2019) as well as Russell and Barley (2020) address—each on a case-specific basis—the challenges of informed consent in longitudinal research approaches and show that informed consent, especially in these longer research processes, cannot be obtained by asking once at the beginning.

Other researchers describe how *bureaucratic consent forms* can lead to problems with gaining rapport (Gabb, 2010; Russell and Barley, 2020) and influence the relationship between participants and researchers (Lillie and Ayling, 2021). Several researchers report that they decided to forego written informed consent and obtain verbal consent for a variety of reasons (protection of participants’ identity, not practical in ethnographic research, and so on) (Gonzalez-Lopez, 2011; Perez, 2019; Plankey-Videla, 2012; Wackenhut, 2018).

In addition to these well-known challenges, researchers reflect on how *framing their research interest* relates to informed consent. They describe how they framed their interest vaguely (McKenzie, 2009) or in such a way that participants and gatekeepers were more willing to consent (Anspach and Mizrachi, 2006; Plankey-Videla, 2012) and thus raise the question of the extent to which participants are adequately informed about their research.

Moreover, ethnographic researchers point out that obtaining informed consent from all participants is not always possible, and only “blurry consent” (Barton, 2011) or non-individual consent, such as “community consent” (Angotti and Sennott, 2015), consent of the group (Paoletti, 2014), “gatekeeper consent” (Plankey-Videla, 2012; similar: Laube, 2021) was obtained. They also reflect that informed consent was not obtained in certain research situations due to covert observation (Elsrud et al., 2016; Ward, 2008). Also, ethnographers describe how they dealt with the non-consent of individuals in the field (McKenzie, 2009; Plankey-Videla, 2012).

A different challenge is *informed consent with the underaged*. Researchers consider how they obtained informed consent from children and adolescents (Gerrard, 2021; Wills et al., 2016), for example, for children growing up without parents (van Dijk, 2015) or whose parents are not reachable (Okyere, 2018). The central theme of these debates is ensuring that consent is appropriate for children and determining whether consent must be obtained from parents or legal guardians.

In addition to researching children, *internet and social media* researchers and *archive researchers* face unique challenges regarding informed consent. For example, researchers write about whether and how informed consent was obtained for different data from the Internet, e.g., social media accounts (Gerrard, 2021) or information about public events (Reich, 2015). Archive researchers describe that they did not need to obtain individual consent to use archival data—in this case, testimonies from Holocaust survivors (Einwohner, 2011). O'Connor and Goodwin (2013) portray challenges of informed consent and a follow-up study based on interviews from an archive.

#### 3.2.2 Ensuring voluntary participation

Voluntary participation as a principle is closely linked to informed consent (American Sociological Association, 2018; British Sociological Association, 2017). Some may argue that voluntary participation is one part of informed consent. We, too, believe that informed consent and voluntary participation are inextricably linked. However, by distinguishing between these two principles, we can address concerns regarding potential coercion more effectively. Yet, as sociologists, we know that social fields are always shaped by structures and social constraints that require scrutiny of voluntariness. There is also a rich discussion about whether and how voluntary participation can be implemented in different research designs. The method of covert observation, for example, seems to conflict with this ethical principle (see, for example Hopf, 2004).

In our sample, a frequently mentioned ethical challenge concerning the voluntariness of participation is the *hierarchical structure of the research field*. Several researchers report that they viewed the voluntariness of the involvement as being at risk because participants may have felt indirectly coerced to participate by their position in the field (“coerced participation,” James, 2013, p. 4). This also happens, for example, when gatekeepers specifically “select” participants. In particular, researchers conducting research in companies (Au and Marks, 2013; Grønning, 1997; Plankey-Videla, 2012) and prisons (Brosens et al., 2015; James, 2013; Meisel, 2008) report the difficulties of ensuring the voluntary nature of participation. For example, inmates may have felt pressured to participate due to the presence of supervisors or correctional officers. Winfield (2022), who conducted research at the United States Military Academy Preparatory School, also reflects whether voluntary participation can be guaranteed in research conducted in an “all-encompassing institution” because of the hierarchical system.

In addition to hierarchy in the field, two researchers reflect on whether *paying participants* affects their voluntary participation. Van Dijk (2015), who conducted a study on children living in a household without parents or other adults in South Africa, chose not to pay money directly to the children. Instead, she tried to establish a reciprocal relationship with the children. Perez (2019) conducted ethnographic research on a group of waste pickers. She paid a small amount of money to the participants she interviewed. As a result, she reports that the participants felt coerced to participate in the interview because the group leader pressured them to give this money to the group’s cash box for alcohol. When this coercion became clear to Perez, she stopped the interviews and did not use the transcripts for the analysis.

Another challenge is *dealing with non-consent.* For example, McKenzie (2009) describes the problem of a person not wanting to participate in an ethnography. Russell and Barley (2020) collected intimate data about third parties through an interview without asking the person’s permission. Both cases illustrate challenges to the voluntariness of participation and demonstrate how closely consent and voluntary participation can be interrelated.

The authors also raise questions about voluntariness in *public and social media research*. When researchers observe public events, it is difficult to obtain informed consent from all participants—the voluntary nature of participation is then not given due to the participants’ lack of knowledge. For example, Barton (2011) describes how she participated in a public event as a researcher and revealed herself as a researcher when people shared personal experiences in small groups. Elsrud et al. (2016) explain that they cannot obtain informed consent from everyone when conducting ethnographic observation at a court hearing. Askanius (2019) researches racist movements, analyzes blog posts, and reflects on ensuring voluntary participation. Likewise, Reich (2015) questions the extent to which informed consent must be obtained when researching further information on the internet about a case in her research.

#### 3.2.3 Avoiding harm

That research participants should not be harmed is universally acknowledged. The literature on research ethics discusses various ways in which harm can occur, such as physiological, legal, social, and psychological (Dixon and Quirke, 2018). Scholars emphasize the complex relationships in which qualitative researchers face ethical dilemmas, for example, when they could harm organizations with their publications while at the same time protecting informants (Fine and Schulman, 2009). In this section, we explore different potential dimensions of harm. It is important to note that not all dimensions have occurred, but the scholars have considered their potential impact.

In our sample, the researchers reflect on the possibility that their research could *reproduce stereotypes* about (marginalized) groups they are investigating and how to avoid them (Barnard, 2005; Britton, 2020; Meisel, 2008; Perez, 2019; Russell and Barley, 2020; Swartz, 2011; Wilson, 2018). Askanius (2019), on the other hand, she raises the question of whether she may indirectly support their work by exploring the narratives of far-right groups. A contrasting challenge is that anonymizing data can *silence (marginalized) voices*, such as those of activists (Guenther, 2009), critical workers (Morrison and Sacchetto, 2018), employees (Paoletti, 2014), or children (Okyere, 2018).

In addition to these dimensions, researchers also point out ethical challenges about *conflicts within their research field*. In some cases, analyzes are not published not to intensify existing frictions (Barton, 2011; Bjørnholt and Farstad, 2014) or to avoid disputes arising (Gonzalez-Lopez, 2011). Researchers also reflect on conflicts arising from their presence in the field (van Dijk, 2015) or their publications (Rupp and Taylor, 2011). Furthermore, Lillie and Ayling (2021), for example, point out that they did not address problematic statements made by participants to avoid a loss of trust (similar challenge: Elliott and Roberts, 2020).

As one of the most mentioned dimensions of harm, researchers point out that *participants’ emotional distress* can occur due to their research (Nash and Moore, 2019; O'Connor and Goodwin, 2013). For example, the research involved topics such as sexualized violence (Barnard, 2005; Gonzalez-Lopez, 2011) or the loss and death of loved ones (van Dijk, 2015). In contrast, researchers rarely write about physical harm and physical altercations (except Baird, 2018).

Finally, researchers also reflect on possible *harm caused by authorities* (see section 9, dealing with deviant actions). Researchers write about (potential) persecution of their participants by state institutions (Mare, 2017; O'Donnell Goldensher, 2022; Scarce, 1994; Wackenhut, 2018; Yusupova, 2019) and negative consequences in the workplace (Tilley and Woodthorpe, 2011).

#### 3.2.4 Anonymizing the data

In most cases, anonymization is a common but complex practice for researchers (Wiles, 2013). It is crucial for the final publication and during the field stay to maintain confidentiality (section 5) and avoid harm (section 3). In the relevant literature, for example, Nespor (2000), on the one hand, problematizes anonymization and also questions damage caused by inadequate anonymization. Vainio (2013), on the other hand, argues that anonymization is worthwhile not only for ethical reasons but also for analytical and ontological reasons. That anonymization leads not only to the protection of the participants but also to a “disappearance” of the research subjects, which has already been addressed in section 3.2.3 (*silence voices*).

In our sample, researchers point out how difficult careful anonymization is (Saunders et al., 2015). Insufficient anonymization can lead to significant ethical problems, as in the case of Stein (2010). *Researchers in organizations*, in particular, draw attention to challenges regarding anonymization. Tilley and Woodthorpe (2011) emphasize that anonymizing case studies in organizations is complex because much contextual information about the cases facilitates de-anonymization. Winfield (2022) and Paoletti (2014) emphasize that internal anonymization in organizational research is challenging because members of the organization can identify other members more easily.

In family (Gabb, 2010) or couples research (Bjørnholt and Farstad, 2014), researchers point out that individuals may *recognize themselves in the data* and that this can lead to problems (e.g., *conflicts*, compare Section 3.2.3). Elliott and Roberts (2020) note that participants can experience the critical interpretations researchers make about their statements.

Also, some data types pose unique challenges for anonymization: Data from *social media* can be easily re-identified through internet searches. Researchers recommend careful anonymization and pseudonymization (Gerrard, 2021; Hennell et al., 2020; Mare, 2017). *Visual data*, such as photographs (Pechurina, 2014; Wills et al., 2016), is also challenging to anonymize.

In some cases in our sample, researchers describe choosing *not to anonymize*. Rupp and Taylor (2011) decided not to anonymize the names of the drag queens with whom they were conducting research because they were public figures. Lillie and Ayling (2021) were researching an elite British school in Nigeria. They obtained permission to use the school’s name because Ayling is a former student, and it would have been easy to identify the school. Finally, in her study of Holocaust survivors, Einwohner (2011) points out that she chose not to anonymize the names of survivors to prevent dehumanization and the repeated loss of identity.

#### 3.2.5 Ensuring confidentiality

In the discussion on research ethics, confidentiality is considered a guiding principle for qualitative researchers and should only be breached in exceptional circumstances (American Sociological Association, 2018; Surmiak, 2020). However, some argue it can hinder transformative science (Baez, 2002) and point out problems with the concept of confidentiality for ethnographic research (Jerolmack and Murphy, 2019). Typically, a distinction is made between internal and external confidentiality: while external confidentiality relates to actors outside the field, internal confidentiality means that actors in the field cannot identify each other (Tolich, 2004). The issue of confidentiality in research is closely linked to other ethical concerns. Maintaining confidentiality protects participants from possible harm, and anonymization is a prerequisite for confidentiality. Therefore, we will not repeat similar challenges such as “silencing voices” or “participants identifying themselves.” Instead, we will focus on two specific study results related to confidentiality.

Researchers in our sample mainly reflect on the difficulties of maintaining *internal confidentiality* (Subramaniam, 2023). For example, in the field of family research, family members may recognize each other in the data (Bjørnholt and Farstad, 2014; Gabb, 2010; Saunders et al., 2015), or they may ask the researchers for information about other family members (van Dijk, 2015). Also, for researchers conducting ethnographic research in various forms of organizations, such as prisons (Nielsen, 2010), hospitals (Paoletti, 2014), factories (Plankey-Videla, 2012), military schools (Winfield, 2022), or prison guard training programs (Ricciardelli, 2022), maintaining internal confidentiality is reported to be particularly challenging.

Another ethical issue researchers reflect on is the *limits of confidentiality.* For example, researchers in adolescent research describe that they would have broken confidentiality when adolescents are at risk, such as through alcohol or drug use. The researchers report that they spoke with their underaged participants about these limits of confidentiality (Hennell et al., 2020; James, 2013; Lohmeyer, 2020).

#### 3.2.6 Managing the relationship between researchers and participants

Discussions of research ethics have traditionally emphasized the relationship between the researchers and those being researched. Feminist researchers like Edwards and Mauthner (2012) argue for an “ethics of care” approach, highlighting the importance of respect and responsibilities in shaping that relationship. Researchers also reflect on the role of trust between the researcher and participants (Guillemin et al., 2016) and the ethical problems that arise from it (Fine, 1993; Ryen, 2010). Therefore, it is unsurprising that researchers in our sample also consider the ethical implications of these relationships and highlight potential challenges. In every research project, the relationships between researchers and researched are unique, making it difficult to summarize them in this category. We, therefore, focus on two key aspects:

In some research, the question of *closeness or distance* to the people being studied may involve ethical issues. Therefore, some researchers in our sample reflect on how being insiders or outsiders affects relationships and ethical challenges in their field (Kremakova, 2014; Mah, 2014). Ricciardelli (2022) reflects on how her close relationship with recruits in an officer training program impacted the participants’ lives. Ramirez-i-Olle (2019) and Rupp and Taylor (2011) reflect on the relationship between friendship and research ethics. Several (primarily female) researchers report *emotional challenges* in their research processes. They describe how they view dynamic opening and support as an ethical practice (Gonzalez-Lopez, 2010, 2011; Shaw, 2011; Shaw et al., 2020; Subramaniam, 2023; Swartz, 2011).

Another challenge is *conflict and negative attitudes* towards participants. Some researchers describe disagreeing with their participants’ views, for example, about alternative healing (Baarts, 2009), political views (Yusupova, 2019), problematic notions of masculinity (Elliott and Roberts, 2020), and chauvinism (Morrison and Sacchetto, 2018), racist attitudes (Mäkinen, 2016) or right-wing initiatives against gay and lesbian rights (Stein, 2010). They describe how these conflicting attitudes and actions affected relationships and how they dealt with them.

#### 3.2.7 Negotiating power asymmetries

The relationship between power and qualitative methodologies, as well as the interplay of epistemology and the positions of researchers in the field, is a widely debated topic in qualitative research (Arendell, 1997; Bourke, 2014; Reich, 2021; Schmitz and Hamann, 2022; Tavory, 2019). Many researchers emphasize the importance of considering power relations in the field for an ethical and reflexive attitude while noting that power is not a rigid and one-sided asymmetrical relationship. Instead, the multilayered and multifaceted nature of power relations in the research process is highlighted (Berger, 2015; Hoffmann, 2007). Some of our previously presented findings already incorporate power as an ethical issue (e.g., informed consent and gatekeepers). We focus on three ethical issues related to power in the research process, which became particularly clear in our literature overview.

Researchers often consider how their *identities and positions* can lead to ethical challenges. For example, differences in class, race, education, and sexual orientation and their intersections influence unequal power relations between researchers and research subjects (Bernstein and Friedman, 2013; Gonzalez-Lopez, 2011; Parvez, 2018; Perez, 2019; Stein, 2010; van Dijk, 2015). Researchers reflect on their privileged positions and the resulting differences they want to overcome. Here, they emphasize that power relations are not to be understood as rigid but can also change during a research process (for example, Parvez, 2018; Perez, 2019; Rupp and Taylor, 2011; Swartz, 2011). Furthermore, the analyzed literature notes that researchers in fields are not consistently and constantly in a privileged position (Barton, 2011; Rupp and Taylor, 2011) but can also be relatively powerless in relation to the participants (Lillie and Ayling, 2021). Power relations, as the different reflections show, are complex and thus lead to specific ethical challenges and “ethically important moments.”

Another ethical issue researchers reflect on is the unequal positions in their *research with children*, some of whom live in precarious conditions. Scholars describe how they can support the children materially or financially and build reciprocal research relationships (Swartz, 2011; van Dijk, 2015).

Moreover, researchers also address unequal power relations that arise in the field. Scholars who conduct their *research in organizations* reflect on unequal positions that lie in the organizational context, e.g., research in prisons (Brosens et al., 2015; James, 2013; Meisel, 2008), military schools (Winfield, 2022), or factories (Morrison and Sacchetto, 2018).

#### 3.2.8 Protecting yourself as a researcher

When discussing research ethics, potential harm to those being researched is often the focus. However, it should also be recognized that researchers can be impacted emotionally, physically, or legally by their research. Some qualitative researchers acknowledge this vulnerability of researchers (Ploder, 2022; Råheim et al., 2016; Sikic Micanovic et al., 2020; Sterie et al., 2023; Traianou and Hammersley, 2024) and suggest protective measures for field research (Grimm et al., 2020). In particular, researchers emphasize the significance of emotions and emotion work in qualitative research (Bergman Blix and Wettergren, 2015; Carroll, 2013; Dickson-Swift et al., 2009; Jackson et al., 2013). In the following section, we will explore different dimensions of risk, such as emotional, legal, physical, and privacy concerns, which researchers reflect on in their publications.

Referring primarily to the work of Hochschild (1983), researchers from a variety of fields indicate that they were *emotionally affected* by their research, felt insecure, or were stressed (Askanius, 2019; Brosens et al., 2015; Gonzalez-Lopez, 2011; Kostet, 2021; Lillie and Ayling, 2021; Lumsden, 2013; Mäkinen, 2016; Meisel, 2008; Nash and Moore, 2019; Plankey-Videla, 2012; Ricciardelli, 2022; Shaw, 2011; Subramaniam, 2023; van Dijk, 2015). The emotional work and involvement in Holocaust research seem particularly noteworthy (Einwohner, 2011; Jacobs, 2004). Considering the researchers’ emotions is thus an essential dimension of ethical practice in our sample.

Other forms of risk for researchers include *legal, physical danger, and sexual assault*. Legal risks matter to researchers; for example, when researchers know about illegal activities (Lumsden, 2013; Ward, 2008) or conducted research in authoritarian contexts (Wackenhut, 2018). Of note is the case of Scarce (1994), who was imprisoned for about 160 days for refusing to share data about his research on the radical environmental movement with the police and maintaining confidentiality. Researchers also report *physical risks*, for example, in the context of gang research (Baird, 2018) or boy racers (Lumsden, 2013). Several female researchers report (possible) sexual harassment and assault in the field (Gonzalez-Lopez, 2011; Lumsden, 2013; Plankey-Videla, 2012).

In addition to these risks, researchers reflect on how to *protect their privacy.* One of the challenges researchers faced was that participants searched for information about them online, including on social media. As a result, it is often reported that they were careful about what information they disclose online to protect their privacy (Gerrard, 2021; Hennell et al., 2020; Reich, 2015; Yusupova, 2019).

#### 3.2.9 Dealing with deviant actions

Studying deviant action is one of the origins of qualitative sociology (Anderson, 2017; Becker, 1997). However, it can lead to ethical challenges (Goode, 2015). In some research projects, it is evident that deviant behavior will be studied since it is the subject of the research. In others, however, deviant actions are unexpectedly observed.

In our analysis, researchers gained knowledge, for example about illegal alcohol consumption by young people (Hennell et al., 2020), drug use (Lohmeyer, 2020; Nielsen, 2010; Rupp and Taylor, 2011), and drug selling (Baird, 2018; Ward, 2008), physical violence (Calvey, 2008), breaking professional and ethical rules in hospitals and institutional settings (Anspach and Mizrachi, 2006), unlicensed home births (O'Donnell Goldensher, 2022), illegal car racing (Lumsden, 2013), or other crimes (James, 2013; Scarce, 1994). Researchers reflect on how a report of deviant actions might have led to (further) criminalization of actors in the field, how their findings could have led to the stigmatization of the studied groups, or how they should have intervened in particular situations. They describe how they dealt with this “guilty knowledge” (Lumsden, 2013) in the research process, an extraordinary ethical challenge.

#### 3.2.10 Conducting covert research

Ethics committees often criticize and treat covert research or observation restrictively due to participants’ lack of informed consent. Some of the most notable ethical controversies in qualitative research, such as the Tea Room Trade study conducted by Laud Humphreys (Babbie, 2004; Humphreys, 1970), revolve around the issue of covert research and deception (Wiles, 2013). As a result, there is an ongoing debate and reflection on this topic, especially in the context of ethnographic research (Iphofen, 2020b; Marzano, 2022). Researchers discuss when to classify research as covert, whether it is always unethical, and whether qualitative researchers can conduct their studies without deception (Fine, 1993; Oliver and Eales, 2008). These questions are also relevant for the scholars in our sample:

Some scholars reflect on their *fully covert research*. From a methodological and epistemological perspective, Calvey (2008) and Ward (2008) argue that their covert research was justified. In his study on nightclubs, Calvey (2008) reports, among other things, a situation in which a female student recognized him, and he then denied it. Thus, in addition to informed consent, other ethical issues may arise during covert research. On the other hand, Elsrud et al. (2016) make ethical arguments for their covert observation. They conducted an ethnography in a court case and wanted to protect the participants’ identities through covert observation.

Not all researchers conduct fully covert research, but they also reflect on *semi-covert*, for example, when they do not always reveal their identity as researchers in public spaces (Bernstein and Friedman, 2013), or only the gatekeepers know about their research project (McKenzie, 2009; Perez, 2019). Laube (2021) describes conducting an open observation but hiding parts of his ethnographic practice in the field, for example, by taking field notes in the restroom, which is spatially separated.

Finally, researchers also reflect on the issue of concealing their identities. Researchers report not revealing parts of their identity (for example, sexual orientation, political views, ethnicity) to gain access to the field (Britton, 2020; Stein, 2010). Hennell et al. (2020) have considered using a false social media profile for field access.

#### 3.2.11 Leaving the field

The act of leaving the research field is an important (Hammersley and Atkinson, 2007, pp. 94–96), but sometimes overlooked matter in qualitative and ethnographic literature. However, it is crucial to carefully plan the exit and consider strategies for staying connected or returning to the field and building enduring relationships (see also section 3.2.6; Gobo and Molle, 2017, pp. 290–297). Moreover, giving back the research findings is sometimes seen as an ethical obligation (O'Mathúna, 2020; Tubaro, 2021). Those two topics—maintaining relationships and sharing their findings—are also present in our sample and discussed as ethical issues.

Some researchers in our sample describe how they *maintained relationships* with participants (Gonzalez-Lopez, 2011; Rupp and Taylor, 2011), and also how they dealt with expectations from the field about the continuing relationship (James, 2013). In contrast, Calvey (2008) intentionally created distance to the field as he quit his covert observation and stopped working as a bouncer.

In addition to maintaining and ending relationships, researchers consider how to *share their findings* with the field (Brosens et al., 2015; Cordner et al., 2012). For this, they used various strategies, for example, encouraging participants to provide feedback on publications (Tilley and Woodthorpe, 2011), giving workshops (Gonzalez-Lopez, 2011), publishing summarized results in the native language of the participants (Guenther, 2009) or making interview scripts available (O'Connor and Goodwin, 2013). Gabb (2010) faced ethical challenges in sharing critical interpretations of findings on family relationships while Ward (2008) admitted to not having the courage to share results.

## 4 Discussion

### 4.1 Summary

Before discussing our study’s limitations, we outline the most important results of the literature review and summarize the ethical challenges that the qualitative sociologists in our sample reflect on. As As we have shown, in our sample it is usually female researchers from the so-called “global north” who publish reflexive accounts. These insights have been published in various journals, particularly since 2010, with criminal and organizational research being the main fields of scrutiny. The ethical challenges researchers reflect on are manifold and arise during all phases of the research process. We identified 11 ethical issues researchers reflect on, which we would like to describe briefly in the following section.

The first five ethical issues we have coded deductively refer to ethical principles found in ethical codices from sociology: (1) Regarding informed consent, researchers emphasize understanding informed consent as an ongoing process. They highlight various challenges related to bureaucratic consent forms, difficulties framing their research interests, dealing with blurry consent, and obtaining informed consent from minors. (2) Scholars point out that *ensuring voluntary participation* is particularly difficult in research fields with hierarchical structures. Additionally, they describe compensating participants, dealing with non-consent, and ensuring voluntary participation in public and social media research as further ethical challenges. (3) Furthermore, researchers illustrate various dimensions of possible *harm*, including reproducing stereotypes, silencing marginalized voices, causing conflicts within research fields, emotional distress, and harm caused by authorities. (4) Furthermore, *anonymizing* data poses significant challenges, especially for scholars conducting research in organizations or working with social media data. Researchers emphasize the risk that participants may be able to recognize themselves in the data, and they also reflect on cases where anonymization is unnecessary. (5) Concerning *ensuring confidentiality*, the researchers primarily discuss challenges in terms of internal and the limits of confidentiality.

In addition to these five issues, which relate to common ethical principles, our inductive coding shows ethical challenges beyond those principles: (6) Managing the *relationship between researchers and participants* is one of these ethical issues. Researchers report that a balance between closeness and distance is essential. Conflicts with the participants and negative attitudes toward the field are also the main challenges regarding this ethical issue. (7) Another crucial issue researchers address is the recognition of *power asymmetries*. The reasons for asymmetries are manifold: asymmetries can arise due to identities and positions when conducting research with vulnerable groups or within organizations. Furthermore, researchers describe how power relations can be changed, reversed, or shaped. (8) Moreover*, protecting yourself as a researcher* is an important ethical issue in our data, encompassing emotional, legal, and physical protection and guarding one’s privacy. (9) Researchers also reflect on *dealing with deviant actions* or “guilty knowledge” that they may observe or gain during the research process. (10) Additionally, researchers consider ethical challenges while *conducting covert research*, whether fully covert, semi-covert, or masking one’s identity. (11) Finally, scholars reflect on how they can maintain relationships and share their results when *leaving the field.*

Our summary shows in line with the methodological literature how diverse and complex ethical challenges can be. Therefore, our results may be helpful for researchers as they provide orientation and an overview about potential challenges. However, it is essential to note that our analysis focuses on a specific set of ethical challenges. Thus, we will address any limitations of our research in the following section.

#### 4.2 Limitations

This study is subject to several limitations. We chose to rely on Web of Science (WoS) as it is considered the most comprehensive source. However, it is not the sole database containing journals focusing on ethical challenges in research. Consequently, the study results present the panorama visible through WoS concerning ethical challenges in qualitative sociology. It becomes evident that the classification employed by WoS significantly influenced all aspects of the results obtained, including ethical challenges and principles. By collecting papers exclusively from the WoS Core Collection through a database search, we neglected other published resources, where researchers reflect on ethical challenges, such as monographs or grey literature. This exclusion means that potentially valuable discussions in these other formats were not considered in our analysis.

Moreover, we focused solely on qualitative research, meaning ethical challenges specific to quantitative or mixed-methods research were not considered. Furthermore, using only the WoS database and only including English publications could be the reason for the intense regional focus on authors from the so-called global north. This regional bias may have led to an underrepresentation of perspectives from the global south, who might face different ethical challenges and have unique approaches to addressing them. The language restriction also means that we cannot generalize our sample.

Additionally, the reliance on WoS classifications means that the way ethical challenges are categorized and indexed in the database directly impacts the study’s findings. While we believe these terms should encompass numerous ethical challenges, there is a possibility that a more comprehensive list could exist to address ethical challenges more distinctly. In our search, besides using general keywords (such as “research ethics” and “ethical dilemma”), we specifically looked for ethical principles (like “confidentiality,” “anonymization,” “informed consent,” and “beneficence”). It is conceivable that not all ethical challenges have been identified due to these limitations, which may have influenced our results.

Furthermore, in our paper, and due to the literature review format, we focused only on the most frequently mentioned issues. This approach meant we could only provide a brief account of the various ethical challenges. We could not present the specific challenges in their context or how the researchers dealt with them. This limitation means that the depth and complexity of ethical issues might not be fully captured.

Nevertheless, we remain confident that our results offer a solid overview of “ethically important moments,” which we aim to discuss and conclude in the following and final section. These issues highlight critical points where ethical considerations are paramount, and we believe our overview contributes to the ongoing discourse on ethical challenges in qualitative sociology.

#### 4.3 Conclusion

The starting point of our paper was the observation of various publications in which researchers reflect on specific ethical challenges they have encountered in their respective projects. Reflexivity, a fundamental characteristic and critical concept of qualitative research, plays a crucial role in shaping the contributions to research ethics in qualitative social research. The broad range of these self-reflective papers on “ethics in practice” can be seen as a part of the “reflexive turn” (Lumsden, 2017; Venkatesh, 2013) in qualitative research and sociology.

Our paper aimed to comprehensively overview crucial ethical challenges in qualitative sociology. Our literature review identified 11 ethical challenges that can provide guidance within the extensive body of literature. The findings on the 11 most important ethical issues are linked to the debate on research ethics in qualitative research. The following will discuss three primary connections to current qualitative research and ethics debates.

First, several authors criticize ethical principalism (Bell and Wray-Bliss, 2011; Blee and Currier, 2011) and argue, for example, that ethical principles are only helpful when “treated as reminders of what ought to be taken into account” (Hammersley, 2015, p. 433). Ethical principles set out in codes of ethics are sometimes like a black box for researchers. It remains unclear in the codes (American Sociological Association, 2018; British Sociological Association, 2017) how these ethical principles are transformed into an “ethical practice” (Guillemin and Gillam, 2004) and what challenges may arise in the process. Our results can be helpful here, as our first five categories show the manifold challenges of bringing ethical principles into practice. Moreover, our results show, in line with the abovementioned literature, that an ethical practice does not end by “following” ethical principles. For example, the ethical issues of *managing relationships between researchers and participants* and negotiating power asymmetries, frequently discussed in the literature, emphasize challenges beyond those principles.

Second, our results also highlight issues discussed in the methodological literature but are not commonly framed as ethical challenges: the question of *leaving the field* on the one hand and the *protection of researchers* on the other. While the first issues remind us (once again) that ethical challenges are present in all phases of the research process, the second issue places the researcher at the center. Moreover, in comparison to Taquette et al.’s (2022) literature review on research ethics, our two categories *dealing with deviant actions* and *conducting covert research* are new. They conducted a literature review about ethical dilemmas in qualitative health researchers. They identified five possible ethical conflicts, namely (1) confidentiality and anonymity, (2) participant’s autonomy, (3) causing damage to participants/researchers/research, (4) mistaking the roles of researcher/therapist/friend, and (5) conflict between researchers who propose qualitative research projects and RECs. We assume that some of the issues highlighted, such as those relating to deviant actions and conducting covert research arise from our focus on sociological research. However, the comparability and accordance of the results suggest that the ethical issues we found are relevant for sociologists and scientists from other disciplines who work with qualitative methods.

Third, our results also refer to ethical issues in specific fields of sociological research. Our paper shows that researchers in *criminology and deviance research*, as well as research in organizations, face particular challenges. In criminology/deviance research, researchers often have “guilty knowledge,” and “covert research” is used more frequently in this field of research. When research is conducted in organizations, the hierarchical structure of the field leads to particular ethical challenges, especially dealing with voluntariness and internal confidentiality.

Researchers will continue to publish reflexively about their ethical practices in the future. New ethical challenges will arise as research fields and qualitative methods constantly evolve. The steady establishment of digital qualitative research, in particular, will raise further ethical questions. We hope that our work and future studies can guide us in navigating the ethical challenges that may arise and help researchers respond appropriately in ethically challenging situations.

## Footnotes

^1^Following databases are included: Science Citation Index Expanded (SCI-Expanded), Social Sciences Citation Index (SSCI), Arts & Humanities Citation (AHCI) and Conference Proceedings Citation (CPCI-S), Conference Proceedings Citation (CPCI-SSH), Emerging Sources Citation (ESCI).

^2^One paper (Subramaniam, 2023) in the sample was published as an online first paper in 2022 and has since been published in the journal. Therefore, we have a paper published in 2023, even though we collected the data in 2022.

^3^For the sections see: https://www.asanet.org/communities-and-sections/sections/current-sections/

^4^In the following, we primarily refer to the codes of ethics of the American Sociological Association (2018) and British Sociological Association (2017), as the researchers in our sample are predominantly located in these countries.
